# Free Appropriate Public Education in the Time of COVID-19

**DOI:** 10.1177/8756870520959659

**Published:** 2020-12

**Authors:** J. Matt Jameson, Sondra M. Stegenga, Joanna Ryan, Ambra Green

**Affiliations:** 1 The University of Utah, Salt Lake City, USA; 2 University of North Dakota, Grand Forks, USA; 3 The University of Texas at Arlington, USA

**Keywords:** rural issues, law and policy, special education, COVID-19

## Abstract

In the spring of 2020, public schools across the United States were forced to close their campuses due to an emerging public health crisis caused by the detection of the first cases of the COVID-19 virus. Although schools closed their buildings, the delivery of educational services did not stop. This included the ongoing provision of services mandated by federal law under the Americans With Disabilities Act (ADA) and the Individuals With Disabilities Education Act (IDEA), which establish educational protections, processes, and rights for students with disabilities and their families to ensure educational equity. In this article, we describe the potential legal implications of COVID-19 for schools, students with disabilities, and their families with a focus on challenges faced in rural areas. Strategies for mitigating legal impacts are described.

In early March 2020, an extremely contagious novel coronavirus, first detected in late 2019 in Wuhan, China, had spread into a global pandemic. Although it is still unclear when COVID-19 began widespread community transmission in the United States, by April 6, 2020, every state had mandated the closure of public school campuses. With the exception of a small number of rural schools in Montana who began opening schools at the discretion of local school boards on May 7, 2020, almost all of these closures were extended through the end of the academic year. Yet, these campus closures were not a complete shutdown of education. Many schools began operating remotely, and a nationwide transition to the remote delivery of instruction was initiated. Through a variety of delivery modalities, teaching and learning continued along with student support services and administrative operations. This necessitated increasing access to technology and broadband internet services to ensure equity and included large-scale efforts to address urban and rural disparities in these critical areas. Public schools faced an additional challenge as, without federal guidance, it was still unclear what schools were required to provide in relation to the federal special education requirements in the time of a pandemic. To provide this guidance, on March 12, 2020, the U.S. Department of Education (USDE) issued a statement relating to remote learning and reaffirming the importance of meeting the mandates of the Individuals With Disabilities Education Improvement Act (IDEA, 2004) in this new remote learning context:

If a [local educational agency, typically a school district (LEA)] continues to provide educational opportunities to the general student population during a school closure [i.e., by providing online learning], the school must ensure that students with disabilities also have equal access to the same opportunities, including the provision of [free appropriate public education (FAPE)]. (34 CFR §§ 104.4, 104.33 (Section 504) and 28 CFR § 35.130 (Title II of the ADA)). [State Educational Agencies (SEAs)], LEAs, and schools must ensure that, to the greatest extent possible, each student with a disability can be provided the special education and related services identified in the student’s [individualized education program (IEP)] developed under [the Individuals with Disabilities Education Act (IDEA)], or a plan developed under Section 504. (34 CFR §§ 300.101 and 300.201 (IDEA), and 34 CFR § 104.33 (Section 504)).

If a child does not receive services during a closure, a child’s IEP team (or appropriate personnel under Section 504) must make an individualized determination whether and to what extent compensatory services may be needed, consistent with applicable requirements, including to make up for any skills that may have been lost.

The equitable provision of educational services for all students is at the forefront of this guidance. Unfortunately, some state education agencies (SEAs) and local education agencies (LEAs) interpreted this to mean that schools should not offer remote learning opportunities for *any* students due to their perceived inability to meet the requirement of the Americans With Disabilities Act (ADA) and the IDEA. As a result, numerous public school students with and without disabilities were not receiving any educational services (Nadworny & Kamenetz, 2020). This prompted the Office of Civil Rights (OCR), the Office of Special Education and Rehabilitative Services (OSERS), and the USDE to issue a memo of clarification to SEAs and LEAs on March 21, 2020. The memo indicated,

A serious misunderstanding . . . has recently circulated within the educational community. As school districts nationwide take necessary steps to protect the health and safety of their students, many are moving to virtual or online education (distance instruction). Some educators, however, have been reluctant to provide any distance instruction because they believe that federal disability law presents insurmountable barriers to remote education. This is simply not true.

To be clear: ensuring compliance with . . . [the ADA and IDEA]. . . should not prevent any school from offering educational programs through distance instruction.

This clarification also provided suggestions to schools as to how they might provide special education accommodations and services in online or other remote formats. The USDE (2020) suggestions included “extensions of time for assignments, videos with accurate captioning or embedded sign language interpreting, accessible reading materials, and many speech or language services through video conferencing” (p. 2). Finally, on April 27, 2020, the USDE Secretary Betsy DeVos clearly stated that the USDE “is not recommending Congress pass any additional waiver concerning the Free Appropriate Public Education (FAPE) and Least Restrictive Environment (LRE) requirements of the IDEA, reiterating that learning must continue for all students during the COVID-19 national emergency” (p. 1).

The expectation was clear. Given some reasonable period of time for the development of a remote learning plan which protects the safety and equity of students and educational service providers, all LEAs would be obligated to provide a free appropriate public education (FAPE) as described by the services and goals/objectives outlined in a student’s IEP (Individualized Education Program). At the center of this is the core legal principle that LEAs cannot simply ignore the academic and individualized support needs of its most vulnerable populations that are protected under federal law. Times of national crisis are not the time to roll back critical civil rights protection. The foundations of these substantive and procedural provisions in the IDEA are built upon the concept of FAPE (and specifically what is an appropriate education) and of the IEP (and 504 plans for students with services provided under the ADA) as a mechanism to ensure IDEA compliance and equitable access to educational supports and services. These important components are described in more detail below.

## The Critical Legal Concepts for COVID-19

### FAPE and the IEP

The concept of FAPE is the cornerstone of IDEA and our nation’s special education law. IDEA asserts that each eligible child with a disability is entitled to a FAPE. It is defined in IDEA (34 CFR §300.17) as an educational program that is individualized to fit the specific needs of a child having a disability or qualifying for special education services. The program must meet the child’s unique needs, provide access to the general education curriculum, and meet state grade-level standards. FAPE emphasizes the importance of special education and related services designed to meet the child’s unique needs and that prepares the child for further education, employment, and independent living. Most importantly for this discussion, FAPE requires special education and related services that are provided in conformity with an IEP that meets the requirements of §300.320 through §300.324 of the IDEA. School districts are considered to be in compliance with the FAPE provisions if the IEP enables the child to achieve meaningful educational progress. It is important to be aware that the definition of meaningful educational progress was being examined through a new lens prior to the COVID-19 national emergency, and the bar had already been raised on what meaningful educational progress constitutes a FAPE.

This change began in 2017, when the United States Supreme Court issued a unanimous opinion in *Endrew F. v. Douglas County School District* (Re-1, 137 S. Ct. 988). In this case, the Court interpreted the scope of the FAPE requirements in the IDEA. The Court overturned the decision of the United States Tenth Circuit Court of Appeals (Tenth Circuit) that Endrew, a public school student with autism who had made almost no progress on his IEP goals, was only entitled to an educational program that was calculated to provide merely more than de minimis educational benefit (i.e., The Rowley Standard). The Supreme Court rejected the Tenth Circuit’s reasoning and ruled that, to meet its substantive obligation under the IDEA, a school must offer FAPE as the intended outcome of a well-designed IEP and moved the bar for “meaningful progress” to be one rooted in demonstrated progress in grade-level academic content. In addition, there is a particular emphasis on parental involvement in defining the individualized educational outcomes through the IEP process. This evolving definition will become an important aspect in determining whether LEAs are meeting the substantive provisions of the IDEA in the time of COVID-19.

## COVID-19 Related Court Cases

Two court cases, both filed on May 19, 2020, relate to the impact of COVID-19 on the education of students with disabilities. They can be used to highlight the critical tensions that are defining FAPE in the time of COVID-19. *Brennan and James v. Wolf, Rivera, and the Pennsylvania Department of Education* is a class action lawsuit brought on behalf of verbal and nonverbal students with autism who use augmentative and alternative communication (AAC) and who are educated in public schools with a teacher to student ratio of not less than one teacher or aide per two students (both Brennan and James had 1:1 staffing ratios). This lawsuit charges that the governor, secretary of education, and the Pennsylvania Department of Education failed to provide the plaintiffs with FAPE. Specifically, the case asserts that the governor failed to identify special education services as “life sustaining” and closed schools. In doing so, schools were left unable to provide FAPE because the limitations of remote learning resulted in the plaintiffs not having their educational needs met “as adequately as the needs of non-disabled children” (p. 12). The case highlights services described in the IEP as critical components of FAPE. The amount of service provided (the plaintiffs went from getting 32.5 hr of week of service in a brick and mortar setting to 1.25 hr per week in the remote learning environment) is an important part of the IEP which requires a description of “the anticipated frequency, location, and duration of . . . services” (20 U.S.C. §14.4(d)(1)(A)(i)(VII)). Changes in the amount of services provided without following the process to review and revise the IEP was in violation of FAPE under IDEA. In addition, the case asserts that the 1:1 staffing ratio is an IEP accommodation that was not being provided, and that students in the plaintiff classes often required “hand over hand” instruction where a trained special educator physically prompts and assists them to complete a task and that this support was “literally impossible to achieve with [remote] education” (p. 18). Finally, the suit also charges that the plaintiffs “have reverted to a lower level of functioning as evidenced by a measurable decrease in skills or behaviors as a result of the closure of schools” (p. 19), and that there was no plan for extended school year (ESY) services as required by the IEP. In essence, by failing to provide the services, supports, and accommodations outlined on students’ IEPs, public schools were failing to provide a FAPE to students with disabilities.

In the second case filed, the *Chicago Teachers Union v. Betsy DeVos; United States Department of Education; the Board of Education of the City of Chicago*, the plaintiffs assert that the Secretary of the USDE, the USDE, and the Chicago Board of Education violated Section 706 of the Administrative Procedure Act (APA) when they withheld a waiver of IDEA regulations (5 U.S.C. §706(1)). They argue that in Section 3511(a) of the Coronavirus Aid, Relief, and Economic Security (CARES) Act, Congress gave Betsy DeVos the authority to waive any regulation under the IDEA or Section 504 of the ADA “if the Secretary determines that such a waiver is necessary and appropriate due to the emergency . . . with respect to COVID-19” (p. 7). By failing to exercise this authority, the suit claims that DeVos acted “arbitrarily and abused her discretion . . . by failing to waive any requirement to redraft tens of thousands of educational plans under [IDEA 34 C.F.R. §300.324(b)(1)(ii)],” the regulations related to reviewing and revising the IEPs for students with disabilities. As a result, Chicago public school teachers would be required to review and revise over 60,000 educational programs and their efforts would be diverted from the “work of teaching and providing services, and as such [would] likely deprive their students of FAPE required by the IDEA” (p. 7). In essence, this case argues that the Chicago teachers did not have the time to transition to remote learning and to review and revise all the IEPs to reflect these changes. Reviewing and updating the large number of IEPs would cause such an administrative or procedural burden that teachers would be unable to provide teaching and supports and would fail to provide FAPE for students with disabilities. Again, the IEP is highlighted as critical in determining the FAPE for students with disabilities.

Both of these court cases focus on the delivery of FAPE and the central role the IEP plays is assuring compliance with the federal law. The IEP is increasingly looked to as the mechanism for students with disabilities to determine what an individual’s FAPE would look like. LEAs need to pay particular attention to the content and process of IEPs as COVID-19 has created an environment where substantive and procedural violations of IDEA are likely to occur. Although the IDEA’s procedural protections are largely contained in the statute, its substantive provisions are generally not well defined and have largely been developed through the courts (e.g., the *Endrew Standard*). A substantive violation arises under the IDEA where the content, such as the educational services contained in the IEP, is insufficient to afford FAPE. Courts have generally viewed IDEA violations as substantive when they involve (a) IEP compliance, (b) the least restrictive learning environment, or (c) the adequacy of the individualized instructions and educational supports contained in an IEP. *Brennan and James v. Wolf* illustrates some of the issues LEAs face in meeting these substantive requirements and providing FAPE in the time of COVID-19.

In contrast, procedural violations occur when an LEA fails to comply with the process-based requirements described in the IDEA. Courts have typically viewed failures to properly carry out the processes for identifying students with disabilities and developing IEPs as procedural violations. More specifically, violations regarding Child Find (i.e., failure to identify students eligible for special education services), and evaluations (discussed in a subsequent section) and violations involving the specific processes for developing IEP services, supports, and goals are viewed as procedural violations. There are several types of protections that apply to the process of developing an IEP, including (a) requirements that parents be involved in the process, (b) that IEP teams have a proper composition, (c) that parents receive notice of changes to an IEP, and (d) that the IEP process be undertaken in a defined period of time. Failure to comply with these protections are typically construed as procedural violations. Both the *Brennan and James v. Wolf* and the *Chicago Teachers Union v. USDE* cases highlight the critical procedural components of the IEP process as being central to the delivery of FAPE in the time of COVID-19. They also illustrate the very real challenges faced by LEAs in meeting the substantive and procedural components of FAPE in the face of a national emergency no one was prepared for. The remainder of this article will outline some of the challenges LEAs face in light of these FAPE requirements with a focus on challenges that are unique to rural and remote schools. We also discuss potential solutions, considerations, and necessary changes for the future to ensure FAPE and promote equity in our educational systems.

## Challenges Facing Rural LEAs

Several challenges emerged as LEAs moved to provide remote educational services for all students following the physical closure of schools during the COVID-19 pandemic. News sources and preliminary research reports suggested these challenges were being addressed in different ways and with varying degrees of success in different areas of the United States. LEAs within rural and remote communities faced additional difficulties, especially as they worked to provide FAPE for students with disabilities.

One challenge confronting LEAs throughout the country since the early weeks of school campus shutdowns was the distribution of and access to technology resources for educators and students to support remote instruction and learning. LEAs had to quickly ensure that educators could deliver remote instructional services for students from their homes or other off-site locations. These efforts often included providing hardware and software resources for staff (i.e., cameras, microphones, computers that could be used remotely, video-conferencing software, instructional software), and attempts to ensure that educators had access to a high-speed internet connection from off-campus locations. At the same time, LEAs needed to consider technology access for students within the communities they served. Just as educators needed hardware, software, and internet connectivity resources to deliver instruction, the students living within LEA communities needed these resources to receive instruction from their homes or other off-campus locations.

A technology gap, or “digital divide,” between non-rural and rural schools and communities in the United States has persisted for decades and has been particularly highlighted during school campus closures. Compared with Americans living in urban or suburban areas of the country, Americans living in rural communities are notably less likely to own a home computer (e.g., desktop or laptop), own a smartphone, or have broadband internet in their homes (Perrin, 2019). Recent studies have found that in rural communities, 24% of adults reported having a major problem accessing the internet, with another 34% reporting at least minor difficulties, suggesting that over half of individuals in rural communities have some difficulty accessing the internet (Parker et al., 2018). These disparities have been attributed both to a lack of community broadband internet infrastructure in rural areas (Levin & Mattey, 2017) and to the comparatively reduced economic resources in these communities (Rideout & Katz, 2016). Although high-speed internet connectivity and access to digital devices for educators and students have been noted issues for LEAs throughout the country during the physical school closure (Dusseault & Pillow, 2020; Editorial Board, 2020), students in rural communities remain particularly vulnerable to the loss of timely and appropriate educational services (Gross & Opalka, 2020).

Many areas of the United States were not able to provide high-quality technology access for rural communities during the school closures and instead relied on low-tech or no-tech instructional delivery systems. News sources reported that some rural educators provided educational services via voice over telephone (Nadworny, 2020), used hard copy exchange of instructional materials through pickup and drop-off sites (Shah, 2020), or chose to travel within communities to provide intermittent face-to-face home instruction for students who could not access technology-based instructional resources from their homes or other off-campus sites (Mitchell, 2020).

Compounding these challenges, LEAs also needed to ensure that families and students within the community could (a) use the technology resources and communication avenues, and (b) access, understand, and support students’ use of the instructional materials. Significant instructional responsibilities fell to families with the closure of schools, including the use of effective instructional strategies for their children with disabilities (Lake, 2020). In some instances, family members were responsible for effectively presenting instructional materials for their children. This required skills that special educators receive years of training and practice to master. In one example, students with extensive support needs who needed physical materials and direct instructional support had instructional materials emailed to their families to print out and then present to the students. The teacher then followed up with parents over email or text after parents delivered instruction (Camera, 2020). This example illustrates some of the issues with technology access and other localized resource disparities that have left many students in rural communities with limited or nonexistent access to materials and effective remote instruction during the campus closures (Gross & Opalka, 2020). Circumstances such as these certainly have implications for equitable delivery of FAPE for students with disabilities in rural areas.

LEAs also had to train educators to use the remote instructional materials and to develop and deliver effective remote instructional procedures. The quick deployment of professional development to support technology-based remote learning was likely an additional challenge for many rural LEAs. Educators in rural school districts have historically received limited and often low-quality professional development related to technology-focused topics, and may lack mentors and professional learning communities to support technology learning (Checovich, 2019). Effective and individualized remote instruction for students with disabilities required another layer of educator training that rural and remote LEAs may have struggled to deliver during the school closures. Rural schools already face a critical shortage of teachers trained to provide specialized educational services for students with disabilities (Rude & Miller, 2018), and often experience constraints (i.e., geographic isolation, few readily accessible specialists) that limit special educators’ access to professional development opportunities that allow them to adapt new instructional practices to meet the needs of their students (Farmer et al., 2018). The shortage of trained personnel and limited access to high-quality professional development likely impacted rural special educators’ ability to rapidly move instruction for students with disabilities to the new remote learning format. Limitations such as these may put rural LEAs at risk of violating the procedural and/or substantive provisions of the IDEA in the time of COVID-19.

The closure of schools also reduced access to critical non-instructional resources for many rural families. In some rural and remote communities, brick-and-mortar schools may be the only source of consistent meals for students (Mitchell, 2020), and families with children with disabilities in these areas may rely on schools for child care as they maintain employment that cannot be conducted remotely (D. Little, 2020). Furthermore, school nurses may serve as the only point of connection between families and health care and community services (Gaines, 2020). The discontinuation of basic services provided by schools may have impacted families’ ability to perform in their increased role in their children’s educational instruction.

Finally, the direct impact of COVID-19 infection on rural and remote communities may affect the equitable provision of FAPE for students with disabilities in these communities. Reduced health and wellness may have left rural families less able to participate in their increased role in the instructional process of their children with disabilities (Clarke, 2014). Many rural communities have limited access to high-quality health care services and information (Garcia et al., 2017). For some families coping with the direct and indirect effects of the pandemic, their children’s access to educational services may have become secondary to more pressing needs, such as maintaining employment for financial solvency or attending to the physical needs of children or other family members. Within this context, some rural communities also coped with increased rates of COVID-19 infection. As the pandemic progressed, it has become increasingly clear that diverse populations in rural areas had a higher risk for contracting the virus. For example, during the second week of June 2020, the Navajo Nation Department of Health reported the highest COVID-19 infection rate in the United States (Mozes, 2020). These high rates of community infection may have caused families in rural and remote areas to, understandably, prioritize basic needs over the support of educational services for their children with disabilities. In light of these realities, we are suggesting LEAs focus on several key strategies to ensure the provision of a FAPE to students with disabilities in the time of COVID-19.

## What Should Schools Do to Meet Individualized Needs in Compliance With the Guidance and Recommendations?

The sudden shift to the remote provision of educational services during COVID-19 has presented challenges to the provision of FAPE for students with disabilities in rural schools. However, there are multiple strategies that may help rural LEAs to ensure that students’ legal rights are met. Historical literature on times of change in special education and research related to the key factors and facilitators of outcomes for students with disabilities identifies a number of strategies foundational to ensuring legal and ethical services for students with or at risk for disabilities (Skrtic & Knackstedt, 2018; Thorius & Maxcy, 2015; Yell et al., 1998). The following sections outline these foundational strategies related to educational rights within the context of the COVID-19 pandemic. Strategies include (a) understanding individualized student and family needs, (b) ensuring authentic family and community partnerships, (c) making data-driven decisions, (d) ensuring the validity of evaluation in online environments, and (e) ensuring research-based strategies for interdisciplinary and interagency collaboration. Table 1 provides a brief summary of these strategies.

**Table 1.** Strategies to Support the Provision of FAPE in Rural LEAs During School Shutdowns.

Strategy	**Examples**
**Understand individualized student and family needs**	
Determine each family’s preferred method of communication	Ask families for their preferred mode of communication (e.g., email, phone, traditional mail) and be willing to use multiple modalities to communicate with families
Determine and address individual and community barriers to remote instruction	Provide a visually supported FAQ document to support remote learning materials Place WiFi hotspots in accessible community locations
Determine and address family health andwellness needs	Arrange regular and ongoing meal pickups for students Provide universal mental health and safety screenings
**Develop partnerships with families and community members**	
Communicate with families in a streamlined, frequent, and consistent way	Provide communication on a regular schedule using each family’s preferred method
Engage families in decision making to meet community needs	Invite families to share issues that they encounter during the physical closure of schools Invite families to propose ideas for resolution of issues and incorporate their ideas when possible
**Use data-driven decision making**	
Maintain or increase the frequency of data collection for each student	Develop user-friendly data sheets for each student, aligned with students’ IEP/IFSP goals and objectives Develop a data collection schedule for each student
Involve families in data collection, if appropriate	Provide families with visually supported directions for providing instruction and collecting data Provide remote or socially distanced family training using recommended safety measures
Consider the content and process of assessment to promote equity	Document the procedures used during assessment for each student Review documented assessment procedures for all students using a best practice checklist
Consider barriers to each student’s access to content and instruction to promote equity	Document procedures used to provide instruction for each student Review documented instructional procedures for all students using a best practice checklist
**Promote ethical and valid evaluation in remote learning environments**	
Continue providing Child Find and educational evaluation services	Provide socially distanced home visits using recommended safety measures Provide online evaluations
Look to guidance from professional organizations	Review the Council for Exceptional Children’s “COVID-19 Concerns for Special Education Administrators” document online Connect with regional and state education service agencies or co-ops for resources, supports, and legislation guidance
Ensure close monitoring and ongoing communication during evaluations	Document and regularly review evaluation procedures for each student
Ensure that families clearly understand their rights related to educational evaluation	Develop visually accessible and easy-to-understand flyers or handouts that list family rights pertaining to special education evaluation Regularly review rights with families
**Promote interagency and interdisciplinary collaboration**	
Use digital resources to establish connections between LEAs and community services agencies	After obtaining family consent, use shared secure cloud-based storage for coordinating meetings and services
Develop trainings to facilitate use of online resources and to promote successful meetings and collaboration	Create and post screencasts or videos that demonstrate the use of shared online platforms Provide training and coaching on strategies for effective collaboration and team meetings

*Note.* FAPE = free appropriate public education; LEAs = local education agencies; FAQ = frequently asked question; IEP = Individualized Education Program; IFSP = Individualized Family Service Plan.

### Determine and Address Individualized Student and Family Needs

The development of IEPs starts with understandingindividualized student and family needs. As schools shift educational practices from in-person to remote learning, they must acknowledge the importance of assessing individualized needs and ensuring that the voices of each student and their families are heard and included in the educational process. This is especially true for teachers in rural schools as these teachers have indicated cultural differences between teachers and the rural community as one reason for attrition (Holme et al., 2017). With an explicit focus on understanding students’ and families’ needs and hearing their voices both individually and collectively, teachers may be better able to understand cultural norms and better individualize to meet educational needs. Educators can gather information from families in a range of ways, including electronically (text or email), through mail, or “face-to-face” with families (including through video-conferencing). Gathering input is essential. Even before COVID-19, many families across a range of demographics reported an increased preference for engagement through alternate forms of communication, such as text, email, or social media, compared with traditional phone calls or on-site meetings (Kraft & Rogers, 2015; Thompson et al., 2015). This makes the natural shift in practices during COVID-19 a unique opportunity to forge new relationships and change communication conventions for engaging families in the educational planning process.

Schools also need to assess the individualized and community-specific barriers to providing adequate remote instruction when creating policies and procedures for online instruction. Rural areas have experienced barriers such as a lack of technology and poor or nonexistent internet access, which limits community-wide access to necessary services and educational supports. Also, issues such as assistance with technology setup and troubleshooting, supports for schoolwork, transportation to pick up needed meals through free and reduced lunch programs, and COVID-19 induced trauma should be considered. Although some barriers may not be easily removed, they can be addressed by individually assessing student and family needs during times of transition. For example, a “Frequently Asked Questions” document written in accessible terms with pictorial prompts can accompany a school issued electronic device or other remote instructional materials. District technical support provider contact information can be provided with this document. The district also may choose to clarify or designate locations or campuses where students could access WiFi hot spot stations and pickup meals from school breakfast or lunch programs while still adhering to safety guidelines. Routine check-in opportunities by educational team members, school counselors, school nurses, social workers, or other school personnel can also be considered. Within each of these procedural decisions, families and the community should be included in the decision-making process to accurately and adequately understand the unique student needs and potential impacts of policy changes. This requires authentic partnerships with families and communities.

### Develop Partnerships With Families and Community Members

Another key strategy for ensuring that LEAs meet their legal obligations and maintain the rights of students and families during a time of significant change is through partnerships with families and communities. Authentic partnerships with families extend beyond sending home materials or messaging to families. These partnerships promote parents, caregivers, and community members as equal participants in the educational process, with shared and meaningful responsibilities (Barr & Saltmarsh, 2014; Coburn et al., 2013). Engagement with families is foundational to improved outcomes for students (Coburn et al., 2013). Family engagement has been associated with increased outcomes in reading, math, and overall academic achievement (Garbacz et al., 2016). Acknowledging these improved outcomes, federal regulations specify the involvement of families in the educational assessment of and planning for students with disabilities across the life span in both Part C serving infants and toddlers with disabilities and Part B of IDEA serving children of school age (IDEA, 2004). Although legislation and research support family engagement, schools rarely facilitate and develop authentic family partnerships (Garbacz et al., 2016).

When families and community members are engaged in educational planning processes and decision making, they are able to contribute to the process in ways that help school personnel to conceptualize FAPE within the unique present and future circumstances of an individual student. Families should be involved in their children’s educational planning and given the opportunity to provide feedback on such issues as school-related activities and compensatory education programming. Providing families and community members with the opportunity to engage in decision-making processes has been linked to increased reports of confidence and competence, ultimately leading to early positive outcomes (Dunst & Dempsey, 2007). Family and community engagement may further help with procedural and substantive compliance in the provision of a FAPE.

COVID-19 has forced schools to shift practices and communicate more with students and families due to the remote provision of services and educational curriculum, providing a unique opportunity to increase interactions and improve relationships with families in the IEP process. To effectively communicate with families, schools should provide communication that is streamlined, frequent, and consistent (Francis et al., 2016). For example, an email, text, or letter (depending on the preference of the family) can be provided once a week on a specified day. Meaningful collaboration and authentic partnerships with families and community members should be primary goals for schools. Rather than just providing information to schools, families and community members should be actively engaged in shaping school practices to meet the unique needs of rural and remote communities. However, these collaborations require policies that also support and promote authentic regular ongoing engagement from families, not just in times of crisis (Green et al., 2018). Ongoing and consistent family and community engagement over time allows for increased trust building and also serves as an early detection system for identifying new needs or concerns in the community as they arise (Francis et al., 2016). For example, many districts began remote services by assessing the technology needs of their stakeholders. This allowed districts to distribute technology resources and establish broadband connectivity through deploying mobile hotspots for some rural areas.

### Use Data-Driven Decision Making

Another way that rural LEAs can help ensure accurate and equitable decision making and FAPE in the educational system is through use of data (Datnow et al., 2013). Despite long-standing legislative requirements and research recommendations, data-driven practices in schools and early childhood systems continue to be an identified area of need in the field of education (J. W. Little, 2012), even during typical educational service delivery. The shift to remote instruction during the COVID-19 pandemic has only compounded this issue. Data collection is a necessary component for assessing students’ academic, behavioral, and socio-emotional progress, and for making objective decisions about students’ educational programming; the frequency at which data are collected must be maintained or even increased (Lane, 2007). Caregivers and families may become involved in data collection through consensus of the IEP team. Family reporting in evaluation and data processes provides value and accuracy to the information upon which students’ educational plans are built (Sheldrick et al., 2012). Garnering family input requires relationship building, authentic partnership with families, and highly trained special education teachers to ensure the substantive and procedural provisions of the IDEA are met.

The content and processes of data collection systems and assessments must be considered when they are used to measure and inform “meaningful educational progress.” Assessment can become an issue of inequity if teachers do not consider barriers to learning and accessing both content and instruction. When teachers are aware of their students’ needs, differentiated instruction (Tomlinson, 2000) and universal design for learning (Hitchcock & Stahl, 2003) can assist in ensuring students’ needs and unique living circumstances are considered when determining educational expectations and outcomes.

For some students, especially students with disabilities such as emotional or behavioral disorders whose characteristics may include anxiety and internalized feelings and emotions, the trauma and cognitive dissonance that may be associated with COVID-19 and related consequences can impede the student’s ability to learn. The impacts of trauma are a known barrier to learning (McInerney & McKlindon, 2014; Sitler, 2009), which may suggest that schools also must pay attention and collect data on students’ internalized behavior during the pandemic. Schools should consider the use of behavior and trauma screeners to identify students who may benefit from social-emotional supports and trauma-informed practices. It is also important to consider the use of universal screeners for assessing other risk factors that may be elevated during the COVID-19 community shutdowns. For example, domestic violence has been reported at increased rates during the pandemic, with children living in 60% of households reporting domestic violence (Campbell, 2020). Community mental health and safety screenings should be considered for all students in rural LEAs during the pandemic, with consideration that children with disabilities are especially vulnerable to abuse and neglect (Lund et al., 2017).

### Promote Ethical and Valid Evaluation in Remote Learning Environments

Related to the importance of assessment and screening in remote environments are the processes of identification and evaluation of students needing special education and early intervention services. Legal obligations related to Child Find start at birth under IDEA Part C regulations. Also, IDEA Part C mandates rapid referral and assessment timelines due to the multiple developmental changes that occur in the first 3 years of life (IDEA, 2004). Referrals, evaluations, and the addition of services and supports for infants and toddlers with or at risk of disabilities must occur year-round. To comply with Part C program timelines, some service providers have conducted evaluations via outdoor, socially distanced home visits using recommended safety measures such as wearing masks and washing hands. Other educational teams have conducted evaluations in online formats through the use of observation measures combined with validated parent report measures (McWilliam, 2020). Evaluation teams in kindergarten to 12th-grade educational settings have worked to gather high-quality existing data for review in eligibility redetermination processes, and have conducted some evaluations in online or socially distanced environments (Council for Exceptional Children [CEC], 2020a). Although these adapted processes have been developed by dedicated teams, researchers, and leaders, the changes may cause issues with standardized assessment validity due to changes in protocol and materials.

Professional organizations, such as the CEC, can provide guidance to rural LEAs concerning special education and early intervention evaluation in remote environments. For example, the authors of a recent CEC publication titled “COVID-19 Concerns for Special Education Administrators” recommend that evaluations continue in remote environments despite concerns about validity (CEC, 2020a). They suggest that there is greater harm in delaying needed educational services than in providing adapted evaluations. However, it is important to acknowledge that assessment conducted in alternate formats must be monitored closely and include ongoing communication with relevant stakeholders. Student rights must be clearly articulated to families, including the options of outside evaluation or re-evaluation if there are concerns about school evaluation outcomes. Special education evaluation is another area where authentic partnerships with families can support accurate data collection, an understanding of the student’s needs and skills, and a consideration of appropriate evaluation processes within the student’s unique living circumstances.

A final consideration related to special education evaluation involves closely monitoring the data and progress of students who received evaluation but did not qualify for special education services. This monitoring may help to ensure that students continue to meet benchmarks in their educational curriculum and that legal obligations are met as students transition back to traditional educational settings and service delivery.

### Promote Interagency and Interdisciplinary Collaboration

Ensuring that every student has access to a high-quality and collaborative team of professionals as mandated by legal obligations of IDEA (2004) should be maintained, despite the shift to online or other remotely delivered educational services. Teaming and collaboration within and across educational and community services can help educational teams to build and implement meaningful educational programs for students (e.g., Bricker et al., 2020; Bruder et al., 2019; Griffiths et al., 2020). During the school shutdowns, it is critical for schools to focus policies on supporting research-based teaming and collaboration practices via technology. Policies should include access to secure cloud formats for creating meeting agendas, sharing notes, viewing and discussing data, and engaging in planning activities. Furthermore, policies regarding training in use of features of online platforms to ensure face-to-face communication can occur regularly for relationship building, sharing and transfer of knowledge, and optimal understanding of communication can also be established. Training in successful meeting techniques can help to ensure efficient and effective use of time, a commodity often in sparse supply due to changing demands and challenges such as consultants and itinerate educational staff who span multiple teams (Splett et al., 2017). The use of effective meeting techniques also ensures that all voices on the team are not only allowed an opportunity to join conversations but are actively encouraged and supported in sharing their knowledge. This is critical to equalizing hierarchies and fostering successful outcomes through the IEP and the delivery of a FAPE (King et al., 2009).

In spite of the many challenges presented by the COVID-19 school shutdowns, these unique times have provided an opportunity for researchers, teachers, and leaders in education to acknowledge critical issues. Recent events have highlighted disparities and amplified critical needs that have gone unaddressed. The rise of these issues has spurred innovation and conversations as schools attempt to begin addressing some of these needs.

## Conclusion

We suggest that special education practices in rural schools mirror the recommendations provided by the CEC (2020b) in regard to service provision and FAPE in the time of COVID-19. First, LEAs must ensure that remote learning and reopening plans are designed to include all students and their families in the planning and implementation discussions and initiatives. If LEAs cannot meet the service and support needs for all students, including those with the most extensive support needs, then providing these services would be unequal under the federal law. In addition, the CEC (2020b) emphasizes that LEAs must prioritize in-person services and schooling for young children and students with disabilities with the most intensive learning and behavioral needs to ensure FAPE. Second, changes to educational supports and services must ensure that vulnerable populations and communities are not put at even more of an increased risk for the community transmission of COVID-19. The impact of the virus’ spread has been shown to be devastating, especially among individuals with disabilities and even more so in rural and remote communities. The efforts to support remote learning must focus on the need for equity of access to supports and services. Student educational outcomes cannot be limited by the number of technology tools they have access to, or the speed of their internet connection. Addressing long-standing disparities in access will be critical to meeting the procedural and substantive provisions of the IDEA. This will be especially true for students with disabilities in rural and remote LEAs across the United States. And third, all service delivery efforts should be designed to preserve the integrity of the IDEA and the substantive and procedural provisions contained in the federal rules and regulations. Only through ongoing authentic engagement with parents and other stakeholders can educational services and supports be developed through the IEP process in such a fashion as to meet unique educational circumstances and provide a means for “meaningful educational progress” and, ultimately, FAPE. Overall, education holds immense potential for breaking cycles of poverty and inequity, ultimately improving education and life outcomes. However, we must first acknowledge our current issues and barriers and create strategies for needed change. This in turn allows us to then move forward together in efforts to ensure a more equitable future for all students.
